# Road dust and its effect on human health: a literature review

**DOI:** 10.4178/epih.e2018013

**Published:** 2018-04-10

**Authors:** Raihan K. Khan, Mark A. Strand

**Affiliations:** 1 Department of Social and Behavioral Health Sciences, School of Public Health, West Virginia University, Morgantown, WV, USA; 2 Department of Public Health, North Dakota State University, Fargo, ND, USA

**Keywords:** Dust, Particulate matter, Review literature as topic, Respiratory system, Risk assessment

## Abstract

The purpose of this study was to determine the effects of road dust on human health. A PubMed search was used to extract references that included the words “road dust” and “health” or “fugitive dust” and “health” in the title or abstract. A total of 46 references were extracted and selected for review after the primary screening of 949 articles. The respiratory system was found to be the most affected system in the human body. Lead, platinum-group elements (platinum, rhodium, and bohrium), aluminum, zinc, vanadium, and polycyclic aromatic hydrocarbons were the components of road dust that were most frequently referenced in the articles reviewed. Road dust was found to have harmful effects on the human body, especially on the respiratory system. To determine the complex mechanism of action of various components of road dust on the human body and the results thereof, the authors recommend a further meta-analysis and extensive risk-assessment research into the health impacts of dust exposure.

## INTRODUCTION

Industrialization of society requires an extension of the road traffic system and urbanization. The production of road dust in urban areas is a consequence of industrialization. Although several studies have investigated the geochemical composition of road dust, relatively few scientific studies have assessed the effects of road dust on human health. This literature review was carried out to summarize the current knowledge of the scientific community on the effects of road dust on human health.

### Definitions

Road dust consists of solid particles that are generated by any mechanical processing of materials, including crushing, grinding, rapid impact, handling, detonation, and decrepitation of organic and inorganic materials such as rock, ore, and metal [1]. When this dust becomes airborne, primarily by the friction of tires moving on unpaved dirt roads and dust-covered paved roads, it is referred to as road dust [1].

Fugitive dust is defined as dust that is not emitted from definable point sources, such as industrial smokestacks. Sources include open fields, roadways, and storage piles [2]. Because of the nature and source of fugitive dust, this paper included both road dust and fugitive dust in the review process.

Particulate matter (PM) refers to mixtures of solid particles and liquid droplets found in the air [3]. Some particles, such as dust, dirt, or smoke, are large or dark enough to be seen with the naked eye. Other forms of PM are smaller, and some are only visible under an electron microscope [3]. PM_10_ refers to inhalable particles with a diameter of 10 μm or smaller, and PM_2.5_ describes fine, inhalable particles that have a diameter of 2.5 μm and smaller.

The hazard index (HI) is “the sum of hazard quotients for substances that affect the same target organ or organ system” [4]. The hazard quotient is “the ratio of the potential exposure to the substance and the level at which there is no expected adverse effect” [5]. The HI is used to indicate only the approximate effects of an agent on an organ system, and it cannot be used to show the probability of adverse effects occurring [5].

**Figure 1.**
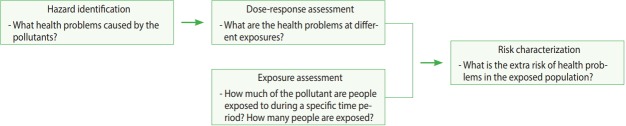
Environmental Protection Agency risk assessment process.

Human health risk assessment is “the process to estimate the nature and probability of adverse health effects in humans who may be exposed to chemicals in contaminated environmental media, now or in the future” [6]. The risk assessment process used by the Environmental Protection Agency (EPA) is graphically shown in Figure 1.

## MATERIALS AND METHODS

### Study selection criteria

A systematic literature review of articles on road dust and its effects on health was carried out. The online search engine of the US National Library of Medicine, PubMed, was used to search scientific journals. Faculty members of North Dakota State University who had a similar interest in the topic also helped to find some of the selected articles. Only articles that met the following criteria were included in the review: (1) Language: Only Englishlanguage articles were selected; (2) Geographical location: Journals from all over the world were considered for the literature review; (3) Sample size: Sample size was not considered in the screening process; (4) Study methodology and statistical analysis: Research methods and the associated statistical analysis were not considered during the screening process; (5) Peer-reviewed journals: Qualifying articles had to be published in a peer-reviewed journal that followed standard methods of peer review; (6) Discussion of health effect: Articles that discussed road dust but did not include any discussion of the effects of road dust on health were excluded from the list; and (7) Keywords: The following keywords were used in various combinations in the literature search using PubMed: road dust and health, fugitive dust and health.

Time limitation: Articles published within 10 years before September 2014 were included in the review process.

**Figure 2.**
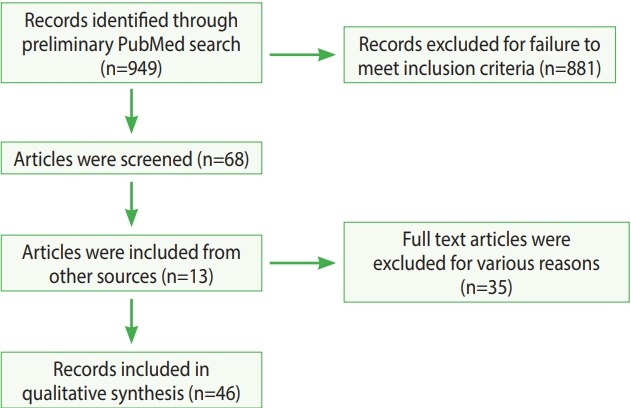
Flow chart of the selection of articles for inclusion in this review.

The primary search yielded 949 results. These results were further screened using the criteria presented above, and finally, a total of 46 articles were selected for the literature review. The process of the entire literature search is illustrated in Figure 2.

### Data extraction and exclusion criteria

The 949 references initially identified using PubMed were distributed between the authors for screening based on the exclusion criteria presented above. References were excluded if they were not from a peer-reviewed journal. Articles that discussed road dust/fugitive dust, but did not discuss the health effects of dust, were excluded from the list. Thirteen articles were collected from faculty members of North Dakota State University who had the same interests and had conducted previous research on road dust. After the initial screening, a thorough and detailed screening excluded 35 more articles that were not related to the health effects of road dust. The study methods, the location of the study area, sample size, and type of statistical analysis were not considered in the exclusion process.

### Review and analysis

The reviewers used Microsoft Excel 2010 (Microsoft Corp., Redmond, WA, USA) to sort the articles. Four columns were placed for each article in each row. The columns were used to sort the articles according to the title, author, potential risks discussed in the articles, and the effect/health hazard of road dust. The articles were randomly distributed to the reviewers. After the initial sorting into the spreadsheet, 2 other spreadsheets were created for further analysis of the results. One spreadsheet was created to analyze the frequency of the diseases/health issues discussed in the articles, and another spreadsheet was created to analyze the frequency of the elements and chemical compounds and radicals that were found in the road dust, along with the corresponding health hazards. Health hazards/issues were sorted according to the organ system. Chemicals were divided into 2 main categories to analyze the frequency of the chemical substances discussed in the articles: elements and compounds.

#### Location of the studies

**Figure 3.**
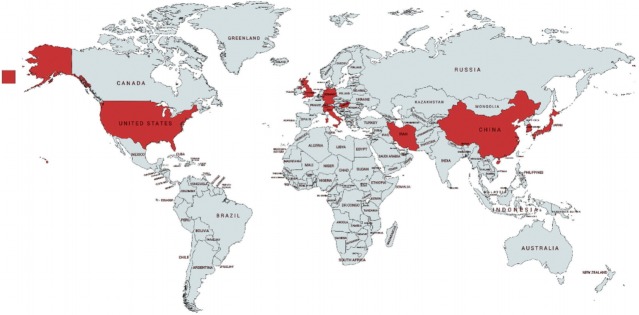
Location of the countries where the reviewed studies were conducted (red color indicates the countries).

The reviewed studies were conducted in multiple countries on 3 continents. A total of 13 studies were carried out in the US, 7 were conducted in the UK, and 7 were carried out in China. One study each was conducted in Germany, Hong Kong, Hungary, Iran, Italy, Japan, Korea, and Switzerland. Figure 3 shows the study locations on a world map. Six studies were review articles that discussed road dust issues.

### Topics discussed by the articles

The studies on road dust had differences in their objectives, study methods, and analyses. Six articles were review articles of studies done on the effects of road dust on human health, while the rest dealt either with the concentration of pollutants in road dust or the effects of road dust on human health. Some articles dealt with both issues. Fourteen studies measured the concentrations of various components of road dust particles and discussed the associated health effects, while clearly indicating the size of the particles that were analyzed. Four studies assessed health effects according to particle size, while 5 did not focus on any specific particle size. One study only measured the concentration of road dust particles, while another study discussed the health effects of road dust in general, without a specific analysis of the concentration of various components of road dust or particle size. The studies used direct and indirect approaches of assessing the health effects of road dust. Studies that used the direct approach collected data either from the study population or secondary data from health databases. In contrast, studies that indirectly assessed health effects either measured the exposure of study population to dust or the health HI. A detailed description of these approaches will follow in the later part of this paper.

### Methods used in the reviewed studies

Various methods were applied to collect dust from the sampling sites. While the majority of the studies collected dust directly from the sampling sites, some studies used reference values for dust matter collected from laboratories. Studies that collected dust from sampling sites used some form of a dust collector to collect and carry the dust to the laboratory for analysis. Eight studies used a plastic hand brush to sweep dust from the sampling site and then used a plastic pan to put the dust inside a plastic bag or container for transport to the laboratory [7-14]. Dust samplers are instruments that contain built-in filters to collect dust of a specific particle size. Six studies used dust samplers of either low-, medium-, or high-volume capacity [15-20]. Faiz et al. [21] used a vacuum cleaner to collect road dust in Pakistan, whereas Campen et al. [22] used a vacuum feeder and Gelencsér et al. [23] used a leaf blower for the same purpose. Table 1 presents a list of dust collectors and samplers employed in the various studies.

We observed the use of different types of filters to filter out the collected dust samples in the laboratories or at the collection site. A Teflon filter was used in 6 studies [15,22,24-27], and a quartz fiber filter was used in 4 studies [15,18,23,27]. Six studies used sieve mesh but did not mention the material used to prepare the mesh [8-11,28,29]. Table 1 presents the filters used in the various studies.

**Figure 4.**
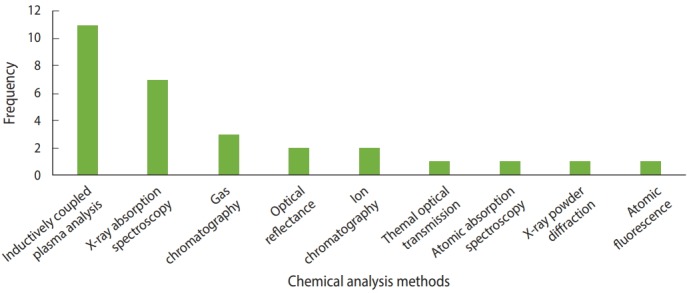
List of chemical analysis methods used.

Various methods were used to analyze the elements and compounds of the dust samples. Inductively coupled plasma (ICP)analysis is a type of plasma emission spectroscopy [35] that was used in 11 studies to analyze the dust samples [7-9,12,17,18,20,30, 36-38]. Several types of ICP methods were used, including ICP-mass spectrometry, ICP-atomic emission spectroscopy, ICP-quadrupole mass spectrometry, ICP-sector-field mass spectrometry, and ICP-optical emission spectrometry. X-ray fluoresence spectroscopy, a form of absorption spectroscopy, was used in 7 studies to analyze the elements that were present [11,15,22-24,28,39]. Four studies used gas chromatography to analyze polycyclic aromatic hydrocarbons (PAHs) [10,14,16,29]. Figure 4 shows the methods of chemical analysis used in the various articles.

While the majority of the studies dealt with PM_2.5_ and PM_10_, some included particles larger than PM_10_. Fifteen studies described working on PM_2.5_ or smaller PM, and 8 of these studies also analyzed PM_10_. Three studies only discussed PM_10_ [15,23,40]. Five studies investigated substances larger than PM_10_ [11-13,21,28].

### Health effects of road dust

Barrett et al. [28] found that lead acetate and lead oxide were more likely to be dissolved in water than other lead compounds due to their high concentration of particles smaller than 38 μm. They reported that insoluble lead compounds were associated with respiratory tract inflammation, which could lead to respiratory tract cancer. Potgieter-Vermaak et al. [11] found that lead and chromium compounds in road dust were present in human body fluids, indicating that exposure to road dust carries certain risks. Lead is known to be responsible for deficits in neurobehavioral and cognitive development in childhood [41]. Reports have also found lead exposure to result in dysfunction of the reproductive system, as well as microcytic anemia resulting in conditions such as hypertension and chronic renal failure [42].

Bell et al. [26] found a significant positive association between PM_2.5_ in road dust and hospital admissions due to cardiovascular and respiratory complications. Ryan et al. [43] found radiographic inflammatory changes in the lung fields of the people living in areas of North Dakota with erionite-containing road dust, which indicated a possible relationship between respiratory inflammation and road dust exposure. Gent et al. [39] found a higher rate of inhaler usage due to increased symptoms among children with asthma who were exposed to fine road dust particles. Kioumourt-zoglou et al. [31] found an association between increased cardiovascular-related hospital admissions and PM_2.5_ in road dust. Mar et al. [33] found a strong association between respiratory symptoms in children and PM_2.5_. Pun et al. [40] determined that PM_10_ from vehicle emissions, nitrate-rich soils, and sea salt was associated with higher rates of hospitalization due to ischemic heart disease in Hong Kong. Campen et al. [22] found that gasoline engine exhaust in road dust was associated with cardiovascular effects in mice.

Franklin et al. [27] established an association between PM_2.5_ and cardiovascular mortality. They also determined that the association was higher in the spring and summer than in the winter. They argued that the presence of certain elements in road dust, such as aluminum and silicon, could modify the association between PM_2.5_ and mortality. Bell et al. [25] found that elements of PM_2.5_ road dust particles such as aluminum and silicon were associated with low birth weight (LBW). They suggested that elemental carbon and zinc from motor vehicle emissions and vanadium and nickel from gasoline burning could also be associated with LBW. Although there is not much evidence of acute or chronic toxicity from zinc or zinc compounds, laboratory tests on mice and other organisms have shown cytotoxicity induced by zinc oxide [44,45]. Aluminum has long been known for its toxic effects on multiple organ systems. Long-term exposure to aluminum was found to be associated with Alzheimer disease [46]. Aluminum was found to be associated with respiratory allergies such as asthma in aluminum industry workers. The accumulation of aluminum can cause cardiac hypertrophy leading to cardiac failure. Aluminum deposition in the body was found to cause inflammation in the hepatobiliary system. Aluminum was also found to be associated with anemia and a low reproductive rate in rats [47].

Colombo et al. [36] established an association between respiratory tract diseases and platinum-group elements in road dust. They reported that the bioavailability of the platinum-group elements depended on their concentration in road dust. Platinum was found to have higher bioavailability in human gastric fluid than palladium or rhodium [37]. Zereini et al. [20] found a higher concentration of platinum-group elements in PM_10_ road dust, and found that palladium had a greater concentration than platinum or rhodium. They reported that palladium was more soluble than other platinum-group elements, thus causing a potential health risk to humans. Farago et al. [38] mentioned the possibility of a mutagenic effect of soluble platinum in road dust that can enter into the human body via water and food. Although metallic platinum has been found to be inert to human health in terms of acute exposure in many toxicological studies, some platinum compounds have been found to cause allergies such as sneezing and wheezing among industrial workers. In laboratory settings, platinum compounds were found to cause carcinoma in mice [48]. Some laboratory experiments on non-human subjects have provided evidence regarding the mutagenicity of some palladium and rhodium salts [49]. Palladium was found to cause allergies at a very low dose among miners, dental technicians, and chemical plant workers [50].

Li et al. [32] measured the health risks of road dust and found that a higher risk was associated with the presence of lead, chromium, and copper in children living near industrial areas. They also found ingestion to be the main route of road dust exposure in humans. Liu et al. [9] found that non-carcinogenic health risks were associated with the presence of higher concentration of barium, lead, and copper in road dust in high-traffic areas. Chromium is known to be carcinogenic. In human subjects, chromium has been found to cause allergic reactions and respiratory distress after short-term exposure. Long-term exposure to chromium has been proven to be associated with lung cancer. In laboratory animals, chromium induced multi-organ carcinoma and birth defects [51]. The health effects of barium have mostly been investigated through experiments on animals, rather than in human studies. In animals, barium causes cardiac arrest, renal failure, anemia, ototoxicity, hepatic failure, infertility, birth defects, and LBW. In humans, most toxic effects have been found to occur through ingestion, which can cause cardiac and renal failure, respiratory arrest, and intestinal bleeding [52]. The health effects of copper on humans are mostly related to the gastrointestinal and hepatobiliary system. Acute copper toxicity can cause gastric upset, nausea, and vomiting. The inhalation of copper can cause al-lergic reactions such as coughing and sneezing, as well as pulmonary fibrosis. Among miners exposed to copper dust, there is evidence of increased vascularity of the mucous membrane inside the nose. Wilson disease is a genetic disorder that leads to accumulation of copper in the liver, resulting in hepatobiliary and renal failure. Increased exposure to copper can aggravate disease progression in Wilson disease patients. In animals exposed to airborne copper or drinking from water with high concentration of copper can lead to immune deficiency [53].

Huang et al. [30] found house air-conditioner dust to be more hazardous than road dust. Zinc was found to have the highest concentration in road dust, while lead was the most abundant in airconditioner dust. They found arsenic to be the riskiest element in their assessment of the risk posed by ingestion and inhalation of these substances. Xu et al. [13] found arsenic to have a higher concentration than other components in road dust near an industrial area, as well as in commercial areas. They found a higher HI for children near industrial plants due to road dust. Additionally, the risk of cancer associated with arsenic exposure via road dust was high in the children. Arsenic has both carcinogenic and non-carcinogenic effects on humans. In a meta-analysis, arsenic was found to be associated with cardiovascular disease, birth defects, neurologic and cognitive disorders, diabetes, ototoxicity, peripheral vascular diseases, and disorders of blood cells such as anemia and leukopenia [54]. That review also found an association between higher levels of arsenic exposure and carcinomas of the skin, liver, lungs, kidney, colon, and urinary bladder [54].

Jiang et al. [29] found higher levels of PAHs than of other compounds in road dust. Their estimated cancer risk of PAH in road dust was higher through the dermal and ingestion routes than through inhalation. They found that children were more susceptible to the hazard than adults due to their greater engagement in hand-to-mouth activities outdoors and lower body weight. Yu et al. [14] found that PAH-contaminated road dust in urban areas was associated with an elevated risk of cancer. They determined that the source of PAHs was a combination of biofuel and coal combustion and traffic engine emissions. Soltani et al. [12] reported high PAH concentrations in road dust near high-traffic roads. They concluded that both adults and children are vulnerable to the potential carcinogenic risk of road dust. In a meta-analysis, evidence was found of an association between PAHs and lung cancer [55]. Ramesh et al. [56] found PAHs to be related to colon cancer and breast cancer in humans, and to show high mutagenicity in laboratory animals.

**Figure 5.**
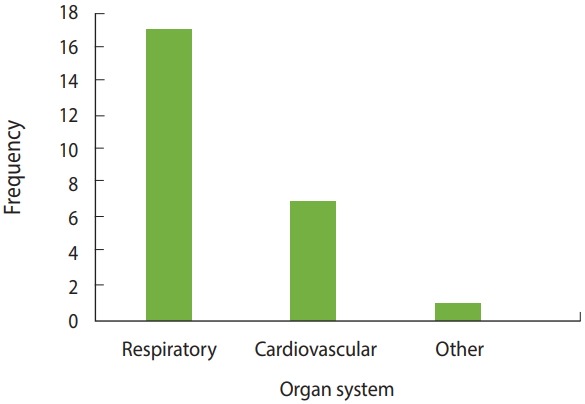
Number of studies mentioning the effects of road dust on various organ systems.

We found 17 studies, including review articles, that reported that exposure to road dust had adverse health effects on the respiratory system. These effects included asthma, as well as forms of respiratory carcinoma such as mesothelioma. Four articles specifically mentioned mesothelioma as one of the effects of road dust on the respiratory system [28,34,57,58]. Seven articles reported that road dust exposure affected the cardiovascular system [18,22, 26,31,40,59,60]. A single study found LBW to be associated with road dust exposure of the mother during pregnancy [25]. Of the 10 articles that indirectly measured health hazards due to road dust exposure, 6 referenced carcinogenicity associated with road dust exposure by calculating the health HI, without mentioning any specific organ system [12-14,21,29,38]. Figure 5 presents a bar graph of the frequency of organ systems referenced in the articles. Table 2 lists the health effects mentioned in the reviewed articles.

**Figure 6.**
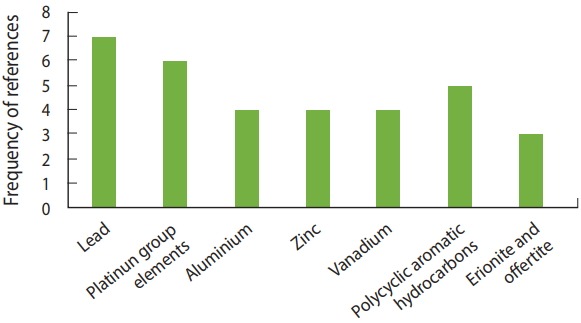
Frequency of chemicals referenced.

Some of the chemical elements discussed in the articles were found to have an association with health hazards. Lead was referenced 7 times. Next, platinum-group elements (platinum, rhodium, bohrium, and palladium) were referenced a total of 6 times. Aluminum, zinc, and vanadium were referenced 4 times each. Among the compounds and radicals, PAHs were referenced a total of 5 times. Natural minerals, such as erionite and offertite found in Turkey and western North Dakota, were referenced 3 times. The other compounds and radicals referenced were nitrogen radicals and oxides and carbon monoxide. Figure 6 shows a bar graph of the frequency of the chemicals referenced in the articles.

### Variations in the research methods of the reviewed articles

While reviewing the studies, it was evident that these studies used markedly different procedures for collecting road dust and analyzing the concentrations of its components. Methods of measuring its health effects also varied.

We found only 15 studies in which researchers directly worked with the study population or used health data collected from hospital registries or local/national health databases to determine the health effects of road dust particles. Bell et al. [25] used birth data collected from the US National Center for Health Statistics, and for another study used Medicare beneficiary files to identify and recruit the at-risk population [26]. Franklin et al. [27] collected non-accidental mortality data from county health offices. Kioumourtzoglou et al. [31] obtained data on emergency hospital admissions for use as the study population. Li et al. [32] obtained data on circulatory disease patients from the China Centers for Disease Control. Farago et al. [38] collected biological samples from 10 study participants. Gatto et al. [16] collected data from 9 volunteers. Gent et al. [39] collected data from 149 children who had been diagnosed with asthma. Kamal et al. [61] analyzed a population sample of 112 children with asthma. Mar et al. [33] recruited 16 adult patients with asthma. Ryan et al. [43] recruited 36 participants. Vedal et al. [60] described 2 studies, one of which recruited 6,814 participants, while the other recruited 93,676 participants. Yamaya et al. [19] worked with 48 study participants.

Ten studies indirectly measured the effects of road dust particles on health. Four of these 10 calculated the health HI [7,9,21,30], while 3 calculated the incremental lifetime cancer risk [13,14,29]. Lorenzi et al. [10] calculated the mean daily intake in micrograms, which is the middle step of calculating the HI. In contrast, Potgieter-Vermaak et al. [11] used a different method known as the Risk Assessment Information System risk calculator. Soltani et al. [12] did not calculate the health HI, but calculated the potential ecological risk index.

### North Dakota’s oil boom and road dust

The first oil boom in North Dakota began in the 1960s. Numerous oil rigs and wells were founded in the following decades in the Bakken formation areas of western North Dakota [62]. The latest oil boom in North Dakota began around 2007 in Mountrail County. According to the US Energy Information Administration, during April 2014 North Dakota and Texas produced 8.4 million barrels per day, which was almost half of the total US crude oil production at that time [63]. A conservative estimation done in 2012 showed that North Dakota’s Bakken region can provide crude oil for up to 30 years [64].

The expansion of the oil industry resulted in high traffic volume on the gravel roads in western North Dakota. It was estimated that in 2014, for every oil well drilled and hydraulically fractured, there were around 2,300 drilling-related truck trips in the Bakken region of North Dakota [65]. In 2014, there were around 190 oil rigs, and 6,800 oil wells were being operated in the Bakken region [66]. High traffic on gravel roads produces road dust, which is a concern for western North Dakota. In a recent study, road dust resuspension was found to be the major source of airborne PM in the Bakken region [66]. According to a report produced by the North Dakota State University Extension Service, local farmers expressed concerns about the effect of road dust on them, their livestock, and their plants [67]. A major concern for western North Dakota is the presence of carcinogenic minerals such as erionite in the rocks of this region [34]. Erionite [68] has been found to cause mesothelioma. However, our literature search did not find any studies of the health effects of the minerals in the Bakken region on its population.

## DISCUSSION

Road dust and its harmful effects on humans are a comparatively new topic for health professionals. Although a few studies have shown a significant association between adverse health effects and road dust, many results were obtained using secondary data, the HI was often determined using laboratory-based extraction methods, and the majority of the health effects were acute. There is evidence of possible adverse health effects on human and laboratory animals for some of the chemicals that were mentioned in the reviewed articles, but none of those studies were done in a situation where the subjects were exposed to dust from multiple sources. Large-scale epidemiological studies in the clinical setting would be the preferred method to identify any direct association between road dust particles and chronic health effects. Debates have emerged regarding the use of single-pollutant-based health assessments or a multi-pollutant-based approach. In recent years, more researchers have supported the use of multidisciplinary, multi-pollutant-based health assessments of road dust exposure to identify which PM sources and chemical compositions are associated with health effects [69]. While some studies showed that road dust particles had an adverse effect on respiratory tract inflammation, such as asthma flare-ups, most of the studies did not measure the direct effects of road dust on other chronic diseases of the respiratory tract, including respiratory carcinoma. The only evidence of respiratory tract carcinoma (mesothelioma) has been reported to be due to the presence of erionite in road dust in villages of Turkey [57]. Erionite and the potentially harmful mineral offertite have been reported to be present in some parts of the US, such as western North Dakota, where people are at risk of exposure to these minerals [34]. Only a single study investigated the effects of road dust on birth outcomes [25]. Due to the teratogenic effects of the elements found in road dust particles, efforts should be made to characterize the teratogenic effects of road dust on pregnant women. Some recent studies recommended considering factors that can influence the distribution and concentration of road dust particles, such as seasonal variations, the presence of industrial plants, and busy traffic areas. These factors contribute to the composition and concentration of road dust PM, and therefore need to be considered when planning for health effect assessment studies [70]. The biological pathways of the effects of road dust particles on humans have not been established. Due to the complexity of identifying biological effects, none of the studies discussed this issue. Source apportionment of the dust particles is another important issue, because researchers should include both exhaust and non-exhaust sources of road dust due to their potential health hazards [71]. Although evidence suggests that rural areas may be likely to have harmful dust particles with significant health hazards [57], almost all studies found a significantly higher concentration of harmful chemicals in urban and industrial areas. Another important finding was the absence of a full 4-step risk assessment in previous studies. While some of the studies assessed the health HI and some measured exposure assessment, none completed the standard EPA risk assessment steps. There were differences in the process of assessing exposure to the chemicals. Moreover, some studies indirectly determined the health HI as part of the exposure assessment instead of the complete 4-step risk assessment process. Additionally, these studies did not use the same chemical standards to measure health effects. Risk characterization is an important part of the risk assessment process that discusses the assumptions, uncertainties, and policy needs regarding the issue in order to assist policy makers. The lack of risk characterization as part of the risk assessment process was a major drawback in the design of the reviewed studies.

### Possible future research

Our literature review presents several opportunities for possible research into the effects of road dust on health. Insufficient research has investigated road dust in rural areas such as western North Dakota. Researchers should work on identifying the possible health effects of the minerals found in the rocks and gravel of western North Dakota. Long-term studies to determine the direct relationship between road dust exposure and chronic health effects, as well as mortality, should be undertaken. Because of advancements in molecular biology, researchers should concentrate on identifying the mechanism of action of road dust particles at the molecular level and seek to identify genetic alterations due to long-term exposure to road dust. There is a lack of research on the effects of road dust during pregnancy, meaning that elucidating the possible teratogenic effects of dust particles on pregnant women is a promising area of research. Seasonal variation in the effects of road dust is another interesting topic that should be prioritized. Researchers should perform the EPA-recommended full 4-step risk assessment for road dust exposure. Especially in regions such as North Dakota, where the oil industry is a major source of road dust, the government should emphasize that oil companies should conduct risk assessments.

### Limitations

There were some limitations to this review process. Only PubMed was used to identify articles. However, the universal acceptability of PubMed in public health and clinical science and the choice of only peer-reviewed articles could compensate for this limitation.

As a comparatively new area of interest in the field of clinical and public health, the effects of road dust on human health are not yet a common concern for researchers. However, the articles that were reviewed raised ample concerns regarding the harmful effects of road dust on health.

## CONCLUSION

This literature review found studies that reported the components of road dust particles to be associated with multiple health effects, in particular on the respiratory and cardiovascular system. The review also found a need for a complete risk assessment of the effects of road dust on human health. We recommend a thorough meta-analysis as well as a 4-step risk assessment process, including a multi-source epidemiological study on road dust particles to identify chronic health effects, with a particular focus on PM_2.5_ and the inclusion of sources in both urban and rural locations.

## Figures and Tables

**Table 1.** List of collectors and filters used

Study	Collector	Filter
Kong et al. [7]	Plastic brush, plastic bag	Polypropylene
Lee et al. [8]	Plastic brush, plastic pan	Sieve mesh
Liu et al. [9]	Plastic brush, plastic bag	Sieve mesh
Lorenzi et al. [10]	Plastic brush, plastic pan	Sieve mesh
Potgieter-Vermaak et al. [11]	Plastic brush, plastic bag	Sieve mesh
Soltani et al. [12]	Plastic brush, plastic bag	Nylon sieve
Xu et al. [13]	Plastic brush, plastic bag	Nylon sieve
Yu et al. [14]	Plastic brush, plastic pan	Nylon sieve
Ducret-Stich et al. [15]	High- and low-volume sampler	Quartz, Teflon
Gatto et al. [16]	Low-volume sampler	Polytetrafluoroethylene
Gómez et al. [17]	Medium-volume sampler	Unknown
Ostro et al. [18]	High-volume sampler	Quartz
Yamaya et al. [19]	High-volume sampler	Unknown
Zereini et al. [20]	High-volume sampler	Cellulose
Faiz et al. [21]	Vacuum cleaner	Steel mesh sieve
Campen et al. [22]	Vacuum feeder	Teflon
Gelencsér et al. [23]	Leaf blower	Quartz
Bell et al. [24]	Unknown	Unknown
Bell et al. [25]	Unknown	Teflon
Bell et al. [26]	Unknown	Teflon
Franklin et al. [27]	Unknown	Nylon, Teflon, quartz
Jiang et al. [29]	Plastic brush, plastic bag	Mesh sieve
Huang et al. [30]	Unknown	Whatman paper
Kioumourtzoglou et al. [31]	Sequential sampler	Unknown
Li et al. [32]	Modified rapid collector	Unknown
Mar et al. [33]	Unknown	Unknown
Saini-Eidukat et al. [34]	Unknown	Vacuum

**Table 2.** List of health effects mentioned in the articles

Organ system	Disease	Frequency
Respiratory	Chronic obstructive pulmonary disease	1
Respiratory	Asthma	6
Respiratory	Fungal infection	1
Respiratory	Deposition in the respiratory tract	4
Respiratory	Allergy	1
Respiratory	Carcinoma	5
Cardiovascular	Emergency cardiovascular disease issues	2
Cardiovascular	Increased mortality due to cardiovascular disease	3
Other	Low birth weight	1
Other	Non-specific carcinoma	7
